# The Influence of Adverse Childhood Experiences on Malevolent Creativity in Young Adulthood

**DOI:** 10.3390/bs13120961

**Published:** 2023-11-22

**Authors:** Natalie A. Ceballos, Toni Terling Watt

**Affiliations:** 1 Department of Psychology, Texas State University, San Marcos, TX 78666, USA; 2 Department of Sociology, Texas State University, San Marcos, TX 78666, USA; tw15@txstate.edu

**Keywords:** adverse childhood experiences (ACEs), coping, empathy, malevolent creativity, social support

## Abstract

Background: Childhood trauma may increase the risk of antisocial behavior in young adulthood. Our study examined the relationship between Adverse Childhood Experiences (ACEs) and the specific antisocial behavior of malevolent creativity (MC), the application of original ideas to purposely harm others, often to gain an unfair advantage through manipulation, threat, or harm. Methods: We surveyed college students (*N* = 524; 78% women) on demographics, ACEs, empathy, social support, coping, general creativity, and malevolent creativity. The data were analyzed via sequential linear regression models. Results: Reporting ≥ 4 ACEs was associated with increased MC, which remained significant when general creativity and demographics were controlled. The association between higher ACEs and MC was no longer significant when psychosocial control variables (social support, empathy, and coping) were included in the statistical model. Social support and empathy were negatively associated with MC, while coping and MC were positively associated. Conclusions: ACEs may increase the likelihood of malevolent creativity in young adulthood, but empathy and social support may disrupt this trajectory. Care should be taken that coping skills, while typically viewed as a positive addition to one’s behavioral repertoire, do not push individuals toward over-reliance on themselves, which may reduce prosocial behaviors and increase MC.

## 1. Introduction

Creative people fill an important role in the world. At their best, they apply their visionary ideas in ways that move society forward. The creative spark is responsible for great works of art and literature, as well as scientific discoveries that have saved millions of lives [1,2]. However, creativity can also have a dark side, which in some cases may lead to individual or societal harm [3,4]. Malevolent creativity is the application of original ideas to purposely harm others, often to gain an unfair advantage through manipulation, threat, or harm [5]. Examples of malevolent creativity may be seen in acts of social manipulation, physical assault, or even terrorism [6]. 

Manifestations of creativity are influenced by the ways in which individuals perceive their world [7]. A person’s life experiences in their environment, both positive and negative, are relevant to their creative processes. Thus, both social information processing theory and life history theory provide relevant theoretical frameworks for the study of malevolent creativity. Social information processing theory suggests that the social information available from one’s environment shapes one’s attitudes, behaviors, and perceptions [8]. Similarly, life history theory posits that humans have developed trade-off strategies based on their environments and that we have applied those strategies through generations to increase our odds of survival in a broad, evolutionary sense [9,10]. Ellis and colleagues [11] apply life history theory at the developmental, rather than evolutionary level, to suggest how the developmental environment (including parental investment, resource scarcity, unpredictability, and so forth) may influence the strategies that individuals will ultimately use as they navigate their environments within their own lifetime, based on the experiences of their pasts. These approaches provide a way of thinking about how an individual might ultimately choose, consciously or unconsciously, to apply their creative spark toward positive or negative ends. 

The study of the relationship between early childhood experiences and adult talent and creativity has a rich, interdisciplinary history. Our research builds on the shoulders of these scholarly giants. In particular, we wish to acknowledge the early groundbreaking work of Goertzel and Goertzel [12]. In the 1960s, this research team published their study of the biographies of eminent personalities, which included analyses of people with positive accomplishments as well as investigations of more notorious individuals. In their research, the Goertzels examined the emotional and intellectual climates in which these eminent individuals were raised. Reviewers of that time note that the Goertzels reached a number of conclusions that challenged traditional beliefs [13]. For example, they described talented and creative people who had experienced childhood trauma and deprivations of the kind that would normally be linked, particularly in the Goertzels’ time, to mental illness or antisocial behavior in adulthood [14]. These biographical techniques continued to be used and refined by later researchers [15]. In addition, transdisciplinary researchers such as Frank Barron acknowledged the inter-relationships between tension, contradiction, disorder, and creative insight, noting that human beings are dynamic systems undergoing constant self-renewal and reorganization [16,17,18,19]. This tradition continues through recent work in the field of neuroscience, which demonstrates that psychological resilience and creativity share a common functional basis in the brain [20]. 

Thus, childhood adversity may have an impact on creative expression throughout the lifespan. The literature on adverse childhood experiences (ACEs) focuses on physical, sexual, and emotional abuse, physical and emotional neglect, and family challenges such as divorce, separation, family mental illness, substance abuse, domestic violence, and family member incarceration [21]. Previous studies suggest that the developmental trajectory may be particularly negatively affected when four or more types of childhood adversity are experienced [21,22,23,24]. Psychoanalytic work suggests that an artist’s traumatic experiences may be consciously or unconsciously evident in their artwork [25], and numerous studies have described the restorative benefits of art therapy for psychological conditions [26]. Further, other work suggests that when creative expression of one type is thwarted, creativity may sometimes go on to manifest in other, darker outlets [27]. 

Previous studies suggest that childhood trauma may increase the risk of engaging in antisocial behavior in young adulthood [28]. However, very few articles have examined the relationship of childhood trauma to malevolent creativity. The existing studies on this topic have exclusively examined Chinese college students as participants. Li and colleagues [29] found that childhood trauma was positively correlated with both aggression and malevolent creativity, while childhood trauma was negatively correlated with resilience. In another study, Jia and colleagues [30] found that positive associations between childhood trauma and malevolent creativity were mediated by dark triad personality factors, particularly in males. Further, Gao and colleagues [31] found that the dark triad personality factors were positively related to malevolent creativity through trait aggression and general creativity. Those authors also speculated that higher moral identity (i.e., one’s desire to maintain a moral image) may serve to prevent individuals from acting on their malevolent tendencies. However, to our knowledge, these relationships have not been examined in college students in the United States (U.S.), nor have they been examined at Hispanic-Serving Institutions (HSIs) in the United States. This is a significant issue, as cultural differences are thought to be related to the perspectives of life history theory and social information processing theory [21].

### Study Hypotheses

The current study examined the relationship between adverse childhood experiences and malevolent creativity in a population of college students at a Hispanic-serving institution in the United States and considered the potential impact of general (positive) creativity, demographics, psychosocial variables, and social support on that relationship. We predicted that a higher level of ACEs would be associated with greater malevolent creativity. The potential impact of demographics and general (positive) creativity on the relationship between ACEs and malevolent creativity was examined as an empirical question. Finally, we hypothesized that the relationship between ACEs and malevolent creativity would be weaker when psychosocial support variables (social support, empathy, and coping) were controlled.

## 2. Materials and Methods

### 2.1. Study Design

Study materials were approved by the participating university’s Institutional Review Board, and participants provided written, informed consent prior to participation. This was a cross-sectional study using self-reported data collected via an anonymous online survey. Questions included information about demographics, ACEs, empathy, social support, coping, general creativity, and malevolent creativity. The data were analyzed via sequential linear regression models.

### 2.2. Participants

College students (*N* = 524; 78% women) were recruited from psychology and sociology courses at a large Hispanic-Serving Institution (HSI) in the South-Central United States using advertisements and word-of-mouth. Participants were compensated with class credit or extra credit.

### 2.3. Measures

#### 2.3.1. Demographics

Participants provided their age, gender, sex, race, ethnicity, and parental education level.

##### Malevolent Creativity

Malevolent creativity was measured using the Malevolent Creativity Behavior Scale (MCBS) [32]. The MCBS is a 13-item scale that measures participants’ malevolent creativity through the activities of their daily lives. It uses a five-point scale. Higher scores indicate a greater frequency of malevolently creative behaviors. Behavioral domains included hurting people (example: how often do you have ideas about how to suppress people who are in your way?), lying (example: how often do you fabricate lies to simplify a problematic situation?), and playing tricks (example: how often do you play tricks on people as revenge?). The coefficient alpha reliabilities for these scales were 0.61 or higher, indicating adequate internal consistency [32].

##### Adverse Childhood Experiences (ACEs)

Participants answered the ten questions from the original ACE study conducted by the Centers for Disease Control and Kaiser Permanente [33]. They were asked, while they were growing up, during their first 18 years of life, did they experience physical, sexual, or emotional abuse, neglect, parents’ divorce, substance use, mental illness, and/or incarceration, or domestic violence? Participants were split into a high ACEs group (ACE score of 4 or higher) and a low ACEs group (ACE score below 4). Previous research reveals ACE scores to be heavily skewed, with very few respondents at the far-right end of the continuum [33,34,35,36]. In addition, research has revealed an important dose-response relationship between ACEs and outcomes, with the most significant elevations in risk occurring for those with four or more ACEs [33,37,38]. This suggests that four or more ACEs represent a tipping point in terms of the allostatic load, or the degree to which the weight of the stress overwhelms the individual’s ability to cope [33,39]. Thus, we believe that it is important to capture this excessive level of strain conceptually rather than treat each different type of adversity as an equal and additive numerical concept. A meta-analysis by Hughes and colleagues [38] of 37 ACE studies revealed that all studies in the review operationalized ACEs in this way. However, to increase the thoroughness of our investigation, we also conducted our analyses with ACEs operationalized as a continuous measure to compare and contrast the different approaches. We do not present these findings in detail but briefly summarize the key findings when they differ from those produced with the dichotomized ACEs measure.

##### General (Positive) Creativity

General (positive) creativity was measured using the Kaufman Domains of Creativity Scale (K-DOCS) [40]. The K-DOCS is a 50-item instrument on which participants are instructed to rate their creativity or creative potential on a variety of tasks using a five-point scale. Creative task domains included self/everyday creativity (example: choosing the best solution to a problem), scholarly creativity (example: coming up with a new way to think about an old debate), performance creativity (example: composing an original song), mechanical/scientific creativity (example: taking apart machines and figuring out how they work), and artistic creativity (example: making a sculpture or piece of pottery). Higher scores indicate greater creativity. The coefficient alpha reliabilities for these scales were 0.83 or higher, indicating good internal consistency [40].

##### Empathy

Empathy was measured using the Interpersonal Reactivity Index (IRI) [41]. The IRI is a 28-item scale composed of statements about one’s thoughts and feelings in a variety of situations, to which participants respond on a five-point scale. The IRI has four subscales: perspective-taking (the ability to adopt the psychological viewpoint of others, essential for non-egocentric behavior), fantasy (the tendency to transpose oneself imaginatively into the feelings and actions of fictitious characters in books, movies, and plays), empathic concern (the experience of other-oriented feelings of sympathy and concern), and personal distress (the endorsement of personal anxiety and unease in tense interpersonal settings) [42]. Higher scores indicate greater empathy. The coefficient alpha reliabilities for these scales were 0.71 or higher, indicating satisfactory internal consistency [42].

##### Social Support

Social support was measured using the Multidimensional Scale of Perceived Social Support (MSPSS) [43,44]. The MSPSS is a 12-item scale that measures participants’ perceived social support from family (example: I can talk about my problems with my family), friends (example: My friends really try to help me), and a significant other (example: There is a special person in my life who cares about my feelings) using a seven-point scale. Higher scores indicate greater social support. The coefficient alpha reliabilities for these scales were 0.90 or higher, indicating excellent internal consistency [44].

##### Coping

The Brief COPE Inventory was used to measure coping [45]. This 28-item inventory assesses a range of ways that people respond to stress, including problem-focused coping and emotion-focused coping, as well as more dysfunctional forms of coping. Higher scores indicate greater use of coping mechanisms. Coefficient alpha reliabilities for these scales ranged from 0.50 to 0.90, indicating adequate internal consistency [45].

### 2.4. Data Analysis Plan

Participant characteristics were described using means (SD) for continuous variables (age) and percentages for categorical variables (gender identity, race/ethnicity, parental education, ACEs group). Next, bivariate associations between ACEs and study variables were examined using independent *t*-tests to compare ACEs groups on the study variables. Finally, in the main analyses of interest, data were analyzed in a series of sequential linear regression models predicting malevolent creativity. The first model examined only ACEs as a predictor. The second model included general (positive) creativity and demographic controls. The third, full model included ACEs, general (positive) creativity, demographics, and psychosocial characteristics (social support, empathetic concern, and coping) as controls. Specific information about these variables is given below.

### 2.5. Sequential Linear Regressions

Dependent Variable. The dependent variable was malevolent creativity. Factor analyses revealed a single factor underlying the malevolent creativity subscales (eigenvalue = 2.178) with factor loadings above 0.66 for each subscale. Regression scoring was used to create a summary measure for malevolent creativity. This approach produces a measure with a mean of zero, where scores above the mean are positive and those below the mean are negative.

Key Independent Variable. Adverse childhood experiences, measured using the ten questions from the original ACE study conducted by the Centers for Disease Control and Kaiser Permanente [33], served as our key independent variable. Research has examined these ACE measures in a number of different ways (e.g., individual items, total score). However, one of the most common techniques for operationalizing adverse childhood experiences is to identify those with an ACE score of 4 or higher [38]. This level of trauma has consistently been associated with significant and substantive increases in the risk of adverse physical and mental health outcomes. Thus, we created a dummy variable to measure having an ACE score of 4 or higher (1 = yes, 0 = no). As noted earlier in Section 2, we also repeated our analyses with ACEs operationalized as a continuous measure so that we could compare and contrast the different approaches.

Control Variables. One of our primary control variables was a measure of general (positive) creativity. We used the five domains from the Kaufman Domains of Creativity to produce a single index capturing creativity [40]. These domains capture self/everyday, performance, scholarly, mechanical/scientific, and artistic creativity. Factor analyses with a scree test revealed a primary underlying factor (eigenvalue = 51.306). Factor loadings for all domains were ≥0.66. Regression scoring was used to create the final index for creativity (mean = 0). We also controlled for several psychosocial traits, which included social support, empathy, and coping. These concepts were measured as continuous variables. Tests of skewness and kurtosis confirm normal distributions for all three psychosocial measures. Finally, we included controls for the following demographic variables: age (years), one or both parents with a college degree (1 = yes, 0 = no), identifying as non-Hispanic white (1 = yes, 0 = no), and identifying as male (1 = yes, 0 = no).

## 3. Results

### 3.1. Participant Characteristics

Table 1 provides a description of the participant sample. The average age of participants was approximately 18 years. The majority of participants identified as female and endorsed a race/ethnicity as other than white. Over half of the sample had one or more parents with a college degree. Close to one-fourth of the sample reported experiencing four or more ACEs.

### 3.2. Bivariate Associations

Table 2 provides the bivariate associations between adverse childhood experiences and all study variables. Results revealed that having experienced four or more adverse childhood events was associated with a significant increase in malevolent creativity but not general (positive) creativity. Respondents in this high ACE category also differed from their peers with lower ACE scores on key psychosocial characteristics. Those with four or more ACEs had lower levels of social support than those with fewer ACEs. However, these respondents reported significantly higher levels of empathetic concern and the use of coping strategies. There were no significant differences in demographic characteristics for those with four or more ACEs compared to those with fewer adverse childhood events. These associations were also found in analyses of ACE scores as a continuous variable.

### 3.3. Sequential Linear Regressions

Table 3 provides a series of sequential linear regression models predicting malevolent creativity. The first model examined only ACEs as a predictor. The second model included general (positive) creativity and demographic controls. The third, full model included ACEs, general (positive) creativity, demographics, and psychosocial characteristics (social support, empathetic concern, and coping). Here, we present our analyses of ACEs, dichotomized into those reporting four or more ACEs compared to those with fewer adverse experiences. However, as noted previously, we also repeated these analyses with the continuous ACE score variable. The key findings were consistent across the two approaches.

Table 3 revealed, as did the bivariate analysis, that respondents with four or more ACEs had significantly higher scores on malevolent creativity as compared to respondents with fewer than four ACEs. This effect remained significant when general (positive) creativity and demographic controls were added. However, the association between having four or more ACEs and malevolent creativity was no longer significant when psychosocial control variables were included in the model. Each of these variables was significantly associated with the dependent variable. Higher levels of social support and empathetic concern were associated with lower levels of malevolent creativity, while higher coping scores were associated with higher levels of malevolent creativity.

## 4. Discussion

While creativity is often viewed as an admirable trait that benefits society, some people use their creativity to malevolent ends. Malevolent creativity is a specific antisocial behavior that involves the application of original ideas to purposely harm others, often to gain an unfair advantage through manipulation, threat, or harm [5]. Because creativity is subjective, it may be influenced by various individual factors, including a person’s social environment and life history. In particular, Ellis and colleagues’ [11] application of life history theory to the developmental trajectory suggests that parental investment, resource scarcity, unpredictability, and other characteristics of a person’s childhood may influence their perceptions and behavior throughout their lifespan. In the current study, we asked whether adverse childhood experiences (defined by whether or not participants had experienced four or more ACEs during their first 18 years of life [38]) were associated with malevolent creativity. Importantly, we considered the potential impact of general (positive) creativity, demographics, psychosocial variables, and social support on that relationship. This study is also innovative in that it is among the first to examine these specific issues among a majority minority population of college students attending a Hispanic-Serving Institution (HSI) in the United States.

First, we predicted that a higher level of adverse childhood experiences would be associated with greater malevolent creativity. Our results supported this hypothesis. We found that participants in the high ACEs group (reporting four or more adverse childhood experiences prior to the age of 18) reported significantly higher levels of malevolent creativity as young adults. This finding aligns with previous studies, which suggested that childhood trauma may be a risk factor for engaging in antisocial behavior in young adulthood [28].

Next, in line with Ellis and colleagues’ application of life history theory, we conducted sequential linear regression analyses to examine the impact of various other factors on the relationship between adverse childhood experiences and malevolent creativity. We first examined the potential impact of demographics and general (positive) creativity on the relationship between adverse childhood experiences and malevolent creativity and found that controlling for these factors did not alter the significance of the relationship between adverse childhood experiences and malevolent creativity. Then, we added controls for psychosocial and social support variables to our statistical model.

In that case, we found that the association between adverse childhood experiences and malevolent creativity was no longer significant. Higher levels of social support and empathic concern were associated with lower levels of malevolent creativity, while higher coping scores were associated with higher levels of malevolent creativity.

These results could imply that there is a path for adverse childhood experiences to *not* lead to malevolent creativity in young adulthood, namely that people who work to harness or enhance their empathy and/or access additional social support, which they lack, may be less likely to apply their creativity to malevolent ends. This is an important point, as people with adverse childhood experiences are often stigmatized, reflecting the common lay perception that childhood abuse results in troubled adults who are unable to adjust properly in social, occupational, or educational situations [46,47,48]. Such stigma can amplify traumatic responses and lead to overreliance on independent coping strategies [47,49,50]. This is consistent with our finding that higher coping scores were positively related to malevolent creativity. Those who are engaged in the development of interventions or therapies for young adults with adverse childhood experiences should be mindful that an overemphasis on coping may push clients toward an overreliance on themselves, which could ultimately reduce prosocial (or increase antisocial) attitudes and behaviors.

### Limitations and Future Directions

Our study supports the notion that a traumatic childhood does not always lead to antisocial behavior such as malevolent creativity in adulthood and suggests avenues by which that pathway may be disrupted. Although the results are intriguing, several issues remain to be addressed in future research. For example, this study relied on self-report and was therefore subject to all the limitations inherent in this approach, including issues related to response bias, social desirability, and memory. We also used a single measure of general (positive) creativity, the Kaufman Domains of Creativity Scale, which limited the number of creative domains that were assessed. Future studies should consider using additional, objective measures for a wider range of creative activities.

It is also important to acknowledge the limitations of the Malevolent Creativity Behavior Scale. Critics of this instrument have suggested that the Malevolent Creativity Behavior Scale focuses primarily on malevolent ideation rather than creativity and have called for the construction of a more valid and reliable method for measuring malevolent creativity, noting that face, construct, and criterion validity could be improved [51]. Though alternative measures remain to be created, these issues should be considered when interpreting our results.

Finally, because life history theory [11] suggests that cultural differences may influence the manifestation of malevolent creativity, it will be important to examine our study questions in a larger and more diverse population with greater variation in race/ethnicity, gender expression, and age. The relationship between stigma and malevolent creativity among those with adverse childhood experiences also warrants further study. Ultimately, a better understanding of malevolent creativity could have implications for a range of environments, from local to global.

## 5. Conclusions

This study contributes to the significant ongoing literature on the influence of early childhood experiences on creativity in adulthood. Although early life adversity may increase the expression of malevolent creativity in young adulthood, the dark side of creativity is not an inevitable destination for young adults with adverse childhood experiences. Our study found that positive psychosocial interventions may interrupt the ACES to malevolence pathway. Specific targets of intervention for individuals with adverse childhood experiences might include work to increase empathy and social support. One way to address this might be through social-emotional learning interventions in children with adverse childhood experiences [52]. We recommend that care be taken to avoid stigmatizing people with adverse childhood experiences and to ensure that coping skills training does not foster an over-reliance on the self, as these factors may ultimately serve to increase trauma and foster malevolent creativity. While this study is not without limitations, it is our hope that these results may increase awareness of malevolent creativity and its precursors, as well as inform future intervention efforts.

## Figures and Tables

**Table 1:** Participant Characteristics.

Variable	Mean (SD) or Percentage	*n*
Age	18.6 (1.2)	524
Gender Identity		
Male	22.5%	118
Female	77.5%	406
Race/Ethnicity		
Non-Hispanic White	36.3%	190
Other	63.7%	334
Parental Education		
College Degree	56.3%	295
No College Degree	43.7%	295
ACE Score		
≥4 ACES	23.1%	120
<4 ACES	76.9%	399

**Table 2:** Bivariate Associations between ACEs and Study Variables.

Variable	ACE Score 4 or Higher	ACE Score < 4	Significance
Malevolent Creativity	0.17 (0.91)	−0.05 (1.03)	*p* < 0.05
General Creativity	0.10 (0.91)	−0.2 (1.01)	*n.s.*
Social Support	60.78 (13.18)	63.77 (14.67)	*p* < 0.05
Empathetic Concern	20.66 (4.84)	19.17 (4.14)	*p* ≤ 0.001
Coping	12.23 (6.53)	10.63 (5.80)	*p* < 0.05
Age	18.6 (1.1)	18.6 (1.2)	*n.s.*

Note: Bivariate analyses were conducted using independent *t*-tests. The term *n.s.* denotes differences that were not statistically significant.

**Table 3:** Sequential Linear Regressions Predicting Malevolent Creativity.

Variable	Model 1	Model 2	Model 3
ACE Score ≥ 4	b = 0.229 *	b = 0.217 *	b = 0.145
	SD = 0.110	SD = 0.110	SD = 0.118
General Creativity		b = 0.119 *	b = 0.151 **
		SD = 0.050	SD = 0.055
Age		b = −0.018	b = −0.021
		SD = 0.044	SD = 0.045
Male		b = 0.220	b = −0.032
		SD = 0.114	SD = 0.124
Non-Hispanic White		b = −0.113	b = −0.053
		SD = 0.103	SD = 0.107
Parental College Degree		b = 0.054	b = 0.068
		SD = 0.100	SD = 0.107
Social Support			b = −0.014 ***
			SD = 0.004
Empathic Concern			b = −0.050 ***
			SD = 0.013
Coping			b = 0.034 ***
			SD = 0.008
R^2^	0.010	0.035	0.169
N	446	446	353

Note: * *p* ≤ 0.05; ** *p* ≤ 0.01; *** *p* ≤ 0.001.

## Data Availability

Data are available upon request from the corresponding author (nc18@txstate.edu).
